# Global profiling of protein lactylation in microglia in experimental high-altitude cerebral edema

**DOI:** 10.1186/s12964-024-01748-x

**Published:** 2024-07-25

**Authors:** Xiufang Jiang, Jiayue Gao, Xuechao Fei, Yanan Geng, Xiangpei Yue, Zibi Shi, Xiang Cheng, Tong Zhao, Ming Fan, Haitao Wu, Ming Zhao, Lingling Zhu

**Affiliations:** 1 Beijing Institute of Basic Medical Sciences, #27 Taiping Road, Haidian District, Beijing, 100850 China; 2 Co-Innovation Center of Neuroregeneration, Nantong University, Nantong, 226019 China; 3 School of Pharmaceutical Sciences, University of South China, Hengyang, 421001 China

**Keywords:** Lactylation, High altitude cerebral edema (HACE), Inflammation, Microglia

## Abstract

**Background:**

High-altitude cerebral edema (HACE) is considered an end-stage acute mountain sickness (AMS) that typically occurs in people after rapid ascent to 2500 m or more. While hypoxia is a fundamental feature of the pathophysiological mechanism of HACE, emerging evidence suggests that inflammation serves as a key risk factor in the occurrence and development of this disease. However, little is known about the molecular mechanism underlying their crosstalk.

**Methods:**

A mouse HACE model was established by combination treatment with hypobaric hypoxia exposure and lipopolysaccharides (LPS) stimulation. Lactylated-proteomic analysis of microglia was performed to reveal the global profile of protein lactylation. Molecular modeling was applied to evaluate the 3-D modeling structures. A combination of experimental approaches, including western blotting, quantitative real-time reverse transcriptionpolymerase chain reaction (qRT-PCR), and enzyme-linked immunosorbent assay (ELISA), confocal microscopy and RNA interference, were used to explore the underlying molecular mechanisms.

**Results:**

We found that hypoxia exposure increased the lactate concentration and lactylation in mouse HACE model. Moreover, hypoxia aggravated the microglial neuroinflammatory response in a lactate-dependent manner. Global profiling of protein lactylation has shown that a large quantity of lysine-lactylated proteins are induced by hypoxia and preferentially occur in protein complexes, such as the NuRD complex, ribosome biogenesis complex, spliceosome complex, and DNA replication complex. The molecular modeling data indicated that lactylation could affect the 3-D theoretical structure and increase the solvent accessible surface area of HDAC1, MTA1 and Gatad2b, the core members of the NuRD complex. Further analysis by knockdown or selectively inhibition indicated that the NuRD complex is involved in hypoxia-mediated aggravation of inflammation.

**Conclusions:**

These results revealed a comprehensive profile of protein lactylation in microglia and suggested that protein lysine lactylation plays an important role in the regulation of protein function and subsequently contributes to the neuroinflammatory response under hypoxic conditions.

**Supplementary Information:**

The online version contains supplementary material available at 10.1186/s12964-024-01748-x.

## Background

High-altitude cerebral edema (HACE), which is characterized by headache, ataxia, fatigue and altered mental status, is a severe form of high-altitude illness that typically occurs after people rapidly ascend to 2500 m or more [1]. It can lead to coma and death without timely diagnosis or management. HACE is considered an end-stage acute mountain sickness (AMS); however, its pathophysiological mechanism is not fully understood [2]. Recently, increasing evidence has demonstrated that inflammation plays critical roles in HACE pathogenesis. An early report showed that hypobaric hypoxia induces an inflammatory response that is positively associated with the development of AMS in humans [3]. Using a mouse HACE model, we previously reported that the lipopolysaccharide (LPS)-induced systemic inflammatory response rapidly exacerbates brain edema upon acute hypobaric hypoxia exposure and eventually impairs neural function [4]. These results indicate that systemic inflammation combined with hypobaric hypoxia exposure could result in increased proinflammatory cytokines and increased blood vessel permeability, thereby leading to vasogenic edema. Sequencing or microarray techniques have revealed that high-altitude exposure can cause changes in the inflammatory profile, and when combined with LPS, it can result in a burst of inflammatory factors [5, 6]. However, the exact mechanism by which hypoxia exacerbates the LPS-induced inflammatory response remains unknown.

In the past few decades, lactate has been recognized as an energy source and metabolic byproduct. However, increasing evidence indicates that lactate serves as a novel signaling molecule in the inflammatory response, neural activity and cancer progression [7, 8]. As a newly identified posttranslational modification, the role of lactate-stimulated lactylation is gradually being recognized. Zhang et al. reported that histone lysine residues serve as epigenetic modifications that directly induce the expression of homeostatic genes [9]. Consistent with these findings, defective lactate production and reduced histone lactylation can blunt the reparative transition [10]. Interestingly, it has also been reported that lactylation of histone proteins can directly drive gene transcription and influence microglial activation [11]. Moreover, the accumulation of nonhistone proteins via lactylation was discovered, and these proteins also contribute to cellular processes. For example, hypoxia induces lactylation in large quantities in microglia and plays pivotal roles in retinal neovascularization [12]. Given that an external hypoxic environment can change the intrinsic oxygen content in tissues [13], it is important to examine lactylation patterns in vivo during exposure to hypoxia and to explore the roles of lactylated proteins in the neuroinflammatory response.

In the present study, we first detected the effect of hypoxia and lactate on the neuroinflammatory response in microglia and explored the induction of the lactylation modification by hypoxia in vitro and in vivo. Then, a proteomic-wide analysis of lactylation was carried out. Additionally, the effects of lactylation on the protein structure were also investigated. These findings will provide new insight into the molecular mechanisms of hypoxia-induced aggravation of neuroinflammation.

## Methods

### Cell culture

The murine BV-2 cell line was obtained from the Cell Resource Center of the Chinese Academy of Medical Sciences (Beijing, China). The cells were cultured in Dulbecco’s minimum essential medium (DMEM) supplemented with 10% fetal bovine serum (FBS) at 37 °C in a humidified atmosphere containing 95% air and 5% CO_2_, after which the cells were split every two days. Cultured primary microglia were isolated from the cerebral cortices of C57BL/6J mice within 1 day after birth. After removing the meninges, the cortical tissues were digested with 0.125% trypsin-EDTA for 30 min at 37 °C, and the reaction was terminated with DMEM/F12 supplemented with 10% fetal bovine serum. The mixed cortical cells were passed through a 70-µm nylon mesh cell strainer and plated on noncoated plastic dishes or plates in DMEM/F12 supplemented with 10% FBS, and the medium was completely replaced every 3–4 days. After reaching confluency at 14 days, the microglia were isolated from mixed glial cultures by shaking at 220 rpm for 2 h in an incubator. The supernatant containing the detached microglia was collected.

### Mouse HACE model

As previously reported, a well-characterized mouse HACE model was established by combining stimulation with LPS (0.5 mg/kg, intraperitoneal injection) and hypobaric hypoxia exposure [4, 14]. Eight-week-old male C57BL/6 mice were purchased from the Laboratory Animal Center of Vital River Experimental Animal Company (Beijing, China). The mice were maintained under specific-pathogen-free (SPF) conditions with a 12-h light/dark cycle at 23 ± 2 °C and free access to standard rodent chow and water. For the hypobaric hypoxia treatment, the mice were placed in a hypobaric hypoxia chamber (model: DYC-DWI; Guizhou, China), and the index was set to mimic an altitude of 6000 m (369.4 mmHg, equal to 10.16% O_2_ at a velocity of 50 m/s for 5 min) for 12 h. The normoxia group was placed in the same chamber set at sea level (100.08 kPa, 20.9% O_2_).

### Lactylated-proteomics analysis

#### Sample preparation and trypsin digestion

Two 10-cm dishes of BV-2 cells were harvested as one sample for the lactylated proteomics assay. The sample was sonicated three times on ice using a high-intensity ultrasonic processor (Scientz) in lysis buffer (8 M urea, 1% protease inhibitor cocktail). The remaining debris was removed by centrifugation at 12,000 × g at 4 °C for 10 min. Finally, the supernatant was collected, and the protein concentration was determined with a BCA kit according to the manufacturer’s instructions. For trypsin digestion, the protein mixture was reduced with 5 mM dithiothreitol for 30 min at 56 °C and alkylated with 11 mM iodoacetamide for 15 min at room temperature in the dark. The protein sample was then diluted by adding 100 mM TEAB (Triethylamonium bicarbonate) to urea at concentrations less than 2 M. Finally, trypsin was added at a 1:50 trypsin-to-protein mass ratio for the first digestion overnight and a 1:100 trypsin-to-protein mass ratio for a second 4 h digestion.

#### HPLC fractionation

After trypsin digestion, the peptides were desalted on a Strata X C18 SPE column (Phenomenex) and vacuum dried. The peptides were reconstituted in 0.5 M TEAB. The sample was then fractionated by high-pH reverse-phase HPLC using an Agilent 300Extend C18 column (5 μm particles, 4.6 mm ID, 250 mm length). Briefly, peptides were first separated with a gradient of 2–60% acetonitrile in 10 mM ammonium bicarbonate (pH 10) over 80 min into 80 fractions.

#### Enrichment of lactylated peptides

To enrich the lactylated modified peptides, tryptic peptides dissolved in NETN buffer (100 mM NaCl, 1 mM EDTA, 50 mM Tris-HCl, 0.5% NP-40, pH 8.0) were incubated with prewashed antibody beads (Cat: PTM-1404, PTM Bio) at 4 °C overnight with gentle shaking. Then, the beads were washed four times with NETN buffer and twice with H_2_O. The bound peptides were eluted from the beads with 0.1% trifluoroacetic acid. Finally, the eluted fractions were combined and vacuum dried.

#### LC-MS/MS analysis

For LC-MS/MS analysis, the resulting peptides were desalted with C18 ZipTips (Millipore) according to the manufacturer’s instructions. The tryptic peptides were dissolved in solvent A (0.1% formic acid, 0.2% acetonitrile in water and directly loaded onto a home-made reversed-phase analytical column (15 cm length, 75 μm i.d.). The gradient comprised an increase in solvent B from 6 to 23% (0.1% formic acid in 98% acetonitrile) over 26 min, 23–35% in 8 min, 80% in 3 min, and 80% for the last 3 min, all at a constant flow rate of 400 nL/min on an EASY-nLC 1000 UPLC system. The peptides were subjected to Capillary source followed by the timsTOF Pro (Bruker Daltonics) mass spectrometry. The applied electrospray voltage was 2.0 kV. The m/z scan range was 350 to 1800 for the full scan, and intact peptides were detected in the Orbitrap at a resolution of 70,000. Peptides were then selected for MS/MS using an NCE setting of 28, and the fragments were detected in the Orbitrap at a resolution of 17,500. A data-dependent procedure alternated between one MS scan and 20 MS/MS scans with 15.0 s dynamic exclusion. The automatic gain control (AGC) was set at 5E4. The fixed first mass was set as 100 m/z.

#### Database search

The resulting MS/MS data were processed using the MaxQuant search engine (v.1.5.2.8). Tandem mass spectra were searched against the Mus_musculus_10090_SP_20201214.fasta database (17,063 entries) concatenated with the reverse decoy database. Trypsin/P was specified as the cleavage enzyme, allowing up to 4 missing cleavages. The mass tolerance for precursor ions was set as 20 ppm in the first search and 5 ppm in the main search, and the mass tolerance for fragment ions was set as 0.02 Da. Carbamidomethyl on Cys was specified as a fixed modification, and lactylation on lysine was specified as a variable modification. The false discovery rate (FDR) was adjusted to < 1%, and the minimum score for modified peptides was set to > 40. The minimum peptide length was set to 7. All the other parameters in MaxQuant were set to the default values. The quality control (QC) validation of the MS data and bioinformatics analysis are presented in Supplementary Text 1.

### Primary microglia sorting with magnetic beads

Mice were treated with a single intraperitoneal injection of LPS (0.5 µg of LPS/g body weight) or with saline as a vehicle control. Twenty-four hours later, the mice were deeply anesthetized with 50 mg/kg sodium pentobarbital and perfused transcardially with ice-cold PBS. The brains were rapidly removed and washed in cold phosphate-buffered saline (PBS). The mouse brain was cut into approximately 8 sagittal slices and transferred to gentle MACS C tubes containing an enzyme mixture (Multi Tissue Dissociation Kit 1, Miltenyi Biotec, Germany). The C Tube was tightly closed and attached upside down to the sleeve of the gentleMACS Octo Dissociator with Heaters, after which the gentleMACS Program 37 C-Multi-F was run. After termination of the program, dissociation of the inflamed neural tissue was continued using a Multi Tissue Dissociation Kit 1 (Miltenyi Biotec, Germany) according to the manufacturer’s protocol. Cell debris was removed from the cell suspension with Debris Removal Solution (Miltenyi Biotec, Germany). Red blood cells were removed from the cell suspension with Red Blood Cell Lysis Solution (Solarbio, Beijing, China). CD11b^+^ cells were positively selected from single-cell suspensions of mouse brain tissue via CD11b (microglia) microbeads (Miltenyi Biotec, Germany). MS columns were used for positive selection and labeling of CD11b^+^ cells. The cells were immediately processed for further applications.

### Western blotting

Total proteins were extracted with RIPA buffer (Applygen, Beijing, China) supplemented with protease inhibitor cocktail (Roche, Mannheim, Germany). A BCA protein assay was used to determine the protein concentration in the supernatant. Equal amounts of protein (20 µg) were separated via sodium dodecyl sulfate-polyacrylamide gel electrophoresis (SDS-PAGE) and electrically transferred onto polyvinylidene difluoride (PVDF) membranes (Millipore, MA, USA). After nonspecific binding was blocked with 5% nonfat milk in Tris-buffered saline/Tween 20 (TBST) at room temperature for 2 h, the membranes were incubated with the following primary antibodies: L-lactyl lysine (Cat#: PTM-1401RM, PTM BIO, Hangzhou), TLR4 (Cat#: ab13556, Abcam), p-IKK (Cat#: 2697, Cell Signaling Technology), IKK (Cat#: 2682, Cell Signaling Technology), p-p65 (Cat#: 3033, Cell Signaling Technology), p65 (Cat#: 8242, Cell Signaling Technology), p-IκBα (Cat#: 2859, Cell Signaling Technology), IκBα (Cat#: 4814, Cell Signaling Technology), pan-acetyl Histone H3 (Cat#: 06599, Sigma-Aldrich), Acetyl-Histone H3 (K9/14) (Cat#: 9677, Cell Signaling Technology), AQP4 (Cat#: 16473-1-AP, Proteintech), ZO-1 (Cat#: ab96587, Abcam), Claudin-5 (Cat#: ab131259, Abcam), Occludin(Cat#: ab216327, Abcam) and β-actin (Cat#: A2228, Sigma‒Aldrich) at 4 °C overnight. After three washes with TBST, the membranes were incubated with an HRP-conjugated secondary antibody diluted at 1:2000 at room temperature for 2 h. After washing three times, the proteins were detected using an enhanced chemiluminescence (ECL) kit (Applygen, Beijing, China).

### Immunofluorescence

The cells were fixed in 4% paraformaldehyde for 15 min at room temperature. After washing with PBS, the cells were permeabilized with 0.1% Triton X-100 for 20 min and continually blocked with 3% BSA (PBS) for 30 min. The cells were then incubated with primary Iba-1 antibody (Cat#: 019–19741, Wako Chemicals) diluted in 3% BSA (PBS) overnight at 4 °C. After washing three times, Alexa Fluor 588/594-labeled secondary antibody (Thermo Fisher Scientific) diluted in PBS were added, and the samples were incubated for 1 h at room temperature in the dark. After washing three times, the cells were mounted in Antifade Mounting Medium containing DAPI (VecorLabs). Images were acquired using a Nikon confocal microscope.

### Quantitative real-time PCR (*q*-PCR)

Total RNA was extracted from microglia using TRIzol Reagent (Thermo Fisher Scientific, Carlsbad, CA) according to the manufacturer’s protocol and quantified using a UV5Nano spectrophotometer (Mettler-Toledo GmbH). First-strand cDNA was synthesized from each sample using an All-in-One First-Strand cDNA Synthesis Kit (GeneCopoeia, Inc., Rockville, MD). Quantitative real-time PCR was performed on a PCR cycler (Bio-Rad CFX96) with synthetic primers (Sangon Biotech, Shanghai). The samples were subjected to the following reaction procedure: 95 °C for 3 min, followed by 45 cycles of 95 °C for 10 s, renaturation for 30 s and 60 °C for 30 s. The 2^−ΔΔCt^ method was used to calculate relative mRNA levels. The expression of β-actin served as an internal control. The sequences of the primers used are shown in the Supplementary Table S1.

### siRNA transfection

LDHA knockdown was achieved by targeted siRNA transfection with Lipofectamine RNAiMAX Transfection Reagent (Thermo Fisher Scientific, Carlsbad, CA) according to the manufacturer’s protocol. BV-2 cells were cultured in 6-well plates at 2 × 10^5^ cells/well for 24 h prior to transfection. On the day of transfection, the medium was replaced with fresh complete medium. Nine microliters of RNAiMAX and 100 pmol of LDHA-siRNA were diluted in 100 µl of Opti-MEM and incubated for 5 min. Subsequently, diluted siRNA solution was added to dilute the RNAiMAX, and the mixture was incubated for 20 min to facilitate the complex formation. The complex was added to each well, and 24 h later, the knockdown efficiency was evaluated via western blotting. The siRNA sequences are shown in Supplementary Fig. S1 and Table. S2.

### Lactate assay

Lactate levels were measured with a lactate assay kit (Jiancheng Bioengineering Institute, Nanjing, China). After treatment, the cell culture supernatant was collected, and the concentrations of lactate in the cell culture medium were measured. To determine the lactate levels in brain tissue, cerebral tissue was lysed in RIPA buffer (with protease inhibitors). After the protein concentration was determined with a BCA assay, the protein concentrations were normalized, and the lactate levels were subsequently measured with a kit.

### Molecular modeling

First, 3D modeling structures of HDAC1, MTA1 and Gatad2b were obtained using the Alpha Fold 2 program (https://github.com/PaddlePaddle/PaddleHelix/tree/dev/apps/paddlefold). Based on the ESFF, the 3-D optimized structures of HDAC1, MTA1 and Gatad2b were modeled based on the steepest descent method. Then, the lactylation modifications of the three proteins were determined using a homology modeling program (InsightII 2000, MSI, San Diego). All calculations were performed on an IBM workstation with a distance-dependent dielectric constant and a long-range nonbonded cutoff of 8 A.

### Statistical analysis

The results are expressed as the mean ± SEM from at least three independent experiments. All the statistical analyses were performed using Prism 7 (GraphPad Software). The mean values from two experimental groups were compared by an unpaired two-tailed Student’s t test. When more than two treatment groups were compared, one-way or two-way ANOVA was used for multiple comparisons in most studies. *p < 0.05, **p < 0.01, and ***p < 0.001 indicated statistical significance.

## Results

### Hypoxia induces lactate production and lactylation

**Fig. 1:**
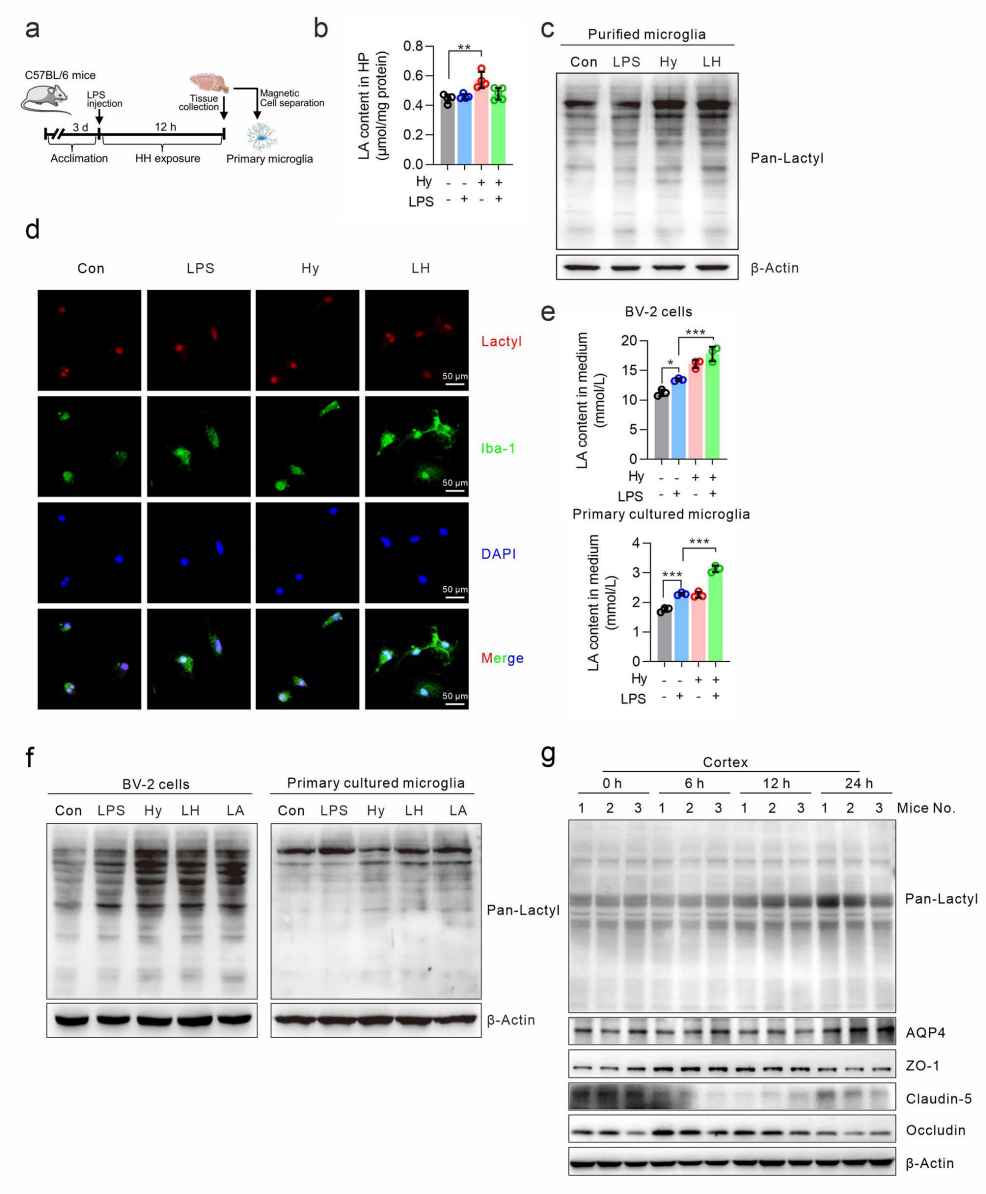
Hypoxia increased the lactate concentration and promoted lactylation. (**a**) The workflow of the mice study. A HACE mouse model was induced by intraperitoneal injection of LPS (0.5 mg/kg) combined with hypobaric hypoxia exposure (mimicking 6000 m) for 12 h. At the end of the treatment, the cerebral tissue was isolated and subjected to magnetic cell isolation to obtain CD11b^+^ primary microglia. (**b**) The lactate concentration in the cerebral tissue of the mice was measured via ELISA. Four mice in each group (**c**) The pan-lactylated proteins in isolated primary microglia were detected via western blotting. LH indicates LPS plus hypoxia, which is the same as below. (**d**) The distribution of lactylated proteins in isolated primary microglia was detected by immunofluorescence with a pan-lactyl lysine antibody. (**e**) BV-2 cells (top) and cultured primary microglia (bottom) were subjected to hypoxia (1% O_2_) and/or LPS (100 ng/ml) for 24 h. The lactate concentration in the culture medium was measured via ELISA. (**f**) The two types of the cells were treated as described above, and the expression of the lactylated proteins was detected with a pan-lactyl lysine antibody. The expression of β-actin served as an internal control

To explore the changes in lactate and lactylation levels in vivo, a mouse HACE model was established as described previously [4, 5], and primary CD11b^+^ microglia were isolated by magnetic cell separation (Fig. 1a). As shown in Fig. 1b, the lactate concentration in the hippocampal region was increased by hypobaric hypoxia or LPS treatment. Western blotting also revealed that lactylation in primary microglia was increased by hypoxia treatment. Moreover, the lactylation levels were greater in the LPS/hypoxia group than in the LPS group (Fig. 1c). Consistent with these findings, the immunofluorescence staining results showed that hypoxia led to an increase in the level of lactylation, which was mainly localized in the nucleus (Fig. 1d). In cultured cell models, we also found that in both cultured BV-2 cells and primary microglia, hypoxia or LPS alone moderately increased the lactate content in the culture medium, but the combination treatment of hypoxia and LPS clearly increased the lactate content (Fig. 1e). Hypoxia or lactate induced an increase in the expression of pan-lactylated proteins, and the lactylated protein levels in the LPS/hypoxia group were markedly greater than those in the LPS group (Fig. 1f). These results demonstrated that hypoxia could increase the lactate content and increase protein lactylation in vivo and in vitro. To explore the potential correlations between protein lactylation and the progression of HACE, the expression of pan-lactylation proteins and HACE protein markers in cortex tissue were examined. As shown in Fig. 1g, the levels of pan-lactylation proteins were increased along with the treatment. Meanwhile, the protein level of AQP4, a water-channel protein expressing highly in brain, was increased, which would facilitate edema fluid formation. Among the tight junction protein, the expression of ZO-1 was firstly increased and then decreased. The expression of Occludin and Claudin-5 were both decreased. Therefore, there were some correlations between the protein lactylation and edema progression in mice HACE model.

### Hypoxia aggravated LPS-induced neuroinflammation in a lactate-dependent manner in microglia

**Fig. 2:**
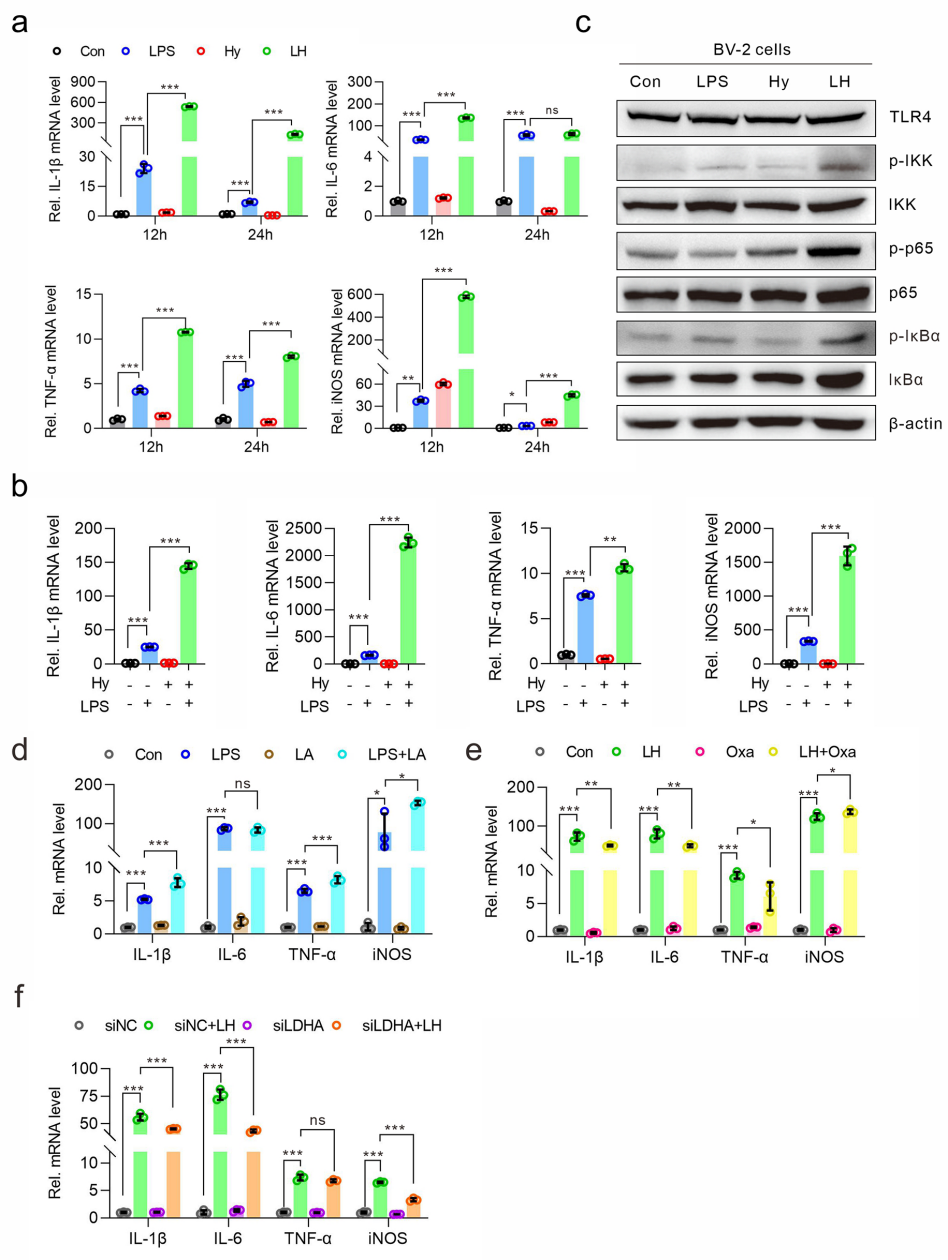
Hypoxia and lactate aggravated microglia-mediated neuroinflammation. (**a**) BV-2 cells were subjected to hypoxia (1% O_2_) or LPS (100 ng/ml) for 12–24 h. The mRNA levels of IL-1β, Il-6, TNF-α and iNOS were detected via *q*-PCR. (**b**) Cultured primary microglia were subjected to hypoxia (1% O_2_) or LPS (100 ng/ml) for 24 h, after which the mRNA levels of inflammatory cytokines were detected via *q*-PCR. (**c**) BV-2 cells were subjected to hypoxia (1% O_2_) or LPS (100 ng/ml) for 24 h, after which the activity of the NF-κB signaling pathway was detected via western blotting. (**d**) BV-2 cells were treated with L-lactate (10 mM) and/or LPS (100 ng/ml) for 24 h. The mRNA levels of inflammatory cytokines were detected via *q*-PCR. (**e**) BV-2 cells were pretreated with the LDH inhibitor oxamate (10 mM) for 1 h and then treated with or without the combination of hypoxia (1% O_2_) and LPS (100 ng/ml) for 24 h. The mRNA levels of inflammatory cytokines were detected by *q*-PCR. (**f**) BV-2 cells were transiently transfected with LDHA-targeted siRNA (siLDHA) or negative control siRNA (siNC) and then treated with a combination of hypoxia (1% O_2_) and LPS (100 ng/ml) for 24 h. The mRNA levels of inflammatory cytokines were detected by *q*-PCR

We then detected the effect of hypoxia on the inflammatory response stimulated by LPS in BV-2 cells. The *q*-PCR results showed that the expression of the proinflammatory cytokines IL-6, IL-1β and TNF-α was obviously greater in the LPS/hypoxia group than in the LPS group. Moreover, the same pattern was observed for the gene expression of iNOS, a typical marker of the M1 phenotype (Fig. 2a). In cultured primary microglia, the expression of cytokines induced by LPS was also aggravated by hypoxia treatment (Fig. 2b). Furthermore, the western blotting results showed that LPS/hypoxia treatment increased the levels of p-IKK, p-p65 and p-IκBα, indicating that the activity of the NF-κB pathway increased (Fig. 2c). These results indicated that hypoxia could aggravate LPS-induced neuroinflammation in microglia. Since hypoxia increases lactate production in microglia, we also detected the role of lactate in the inflammatory response in BV-2 cells. The *q*-PCR results showed that, similar to hypoxia treatment, lactate aggravated the LPS-induced increase in cytokine expression (Fig. 2d). Moreover, when the production of lactate was blocked by the specific inhibitor sodium oxamate, the LPS/hypoxia-induced increase in cytokine expression was inhibited (Fig. 2e). In addition, the LPS/hypoxia-induced expression of cytokines was inhibited by the knockdown of the LDHA protein (the knockdown efficiency of LDHA is shown in Supplementary Fig. S1), an enzyme that catalyzes the production of lactate from pyruvate (Fig. 2f). These results demonstrated that lactate plays an important role in the neuroinflammatory response under hypoxic conditions.

### Identification and analysis of lysine-lactylated sites and proteins

**Fig. 3:**
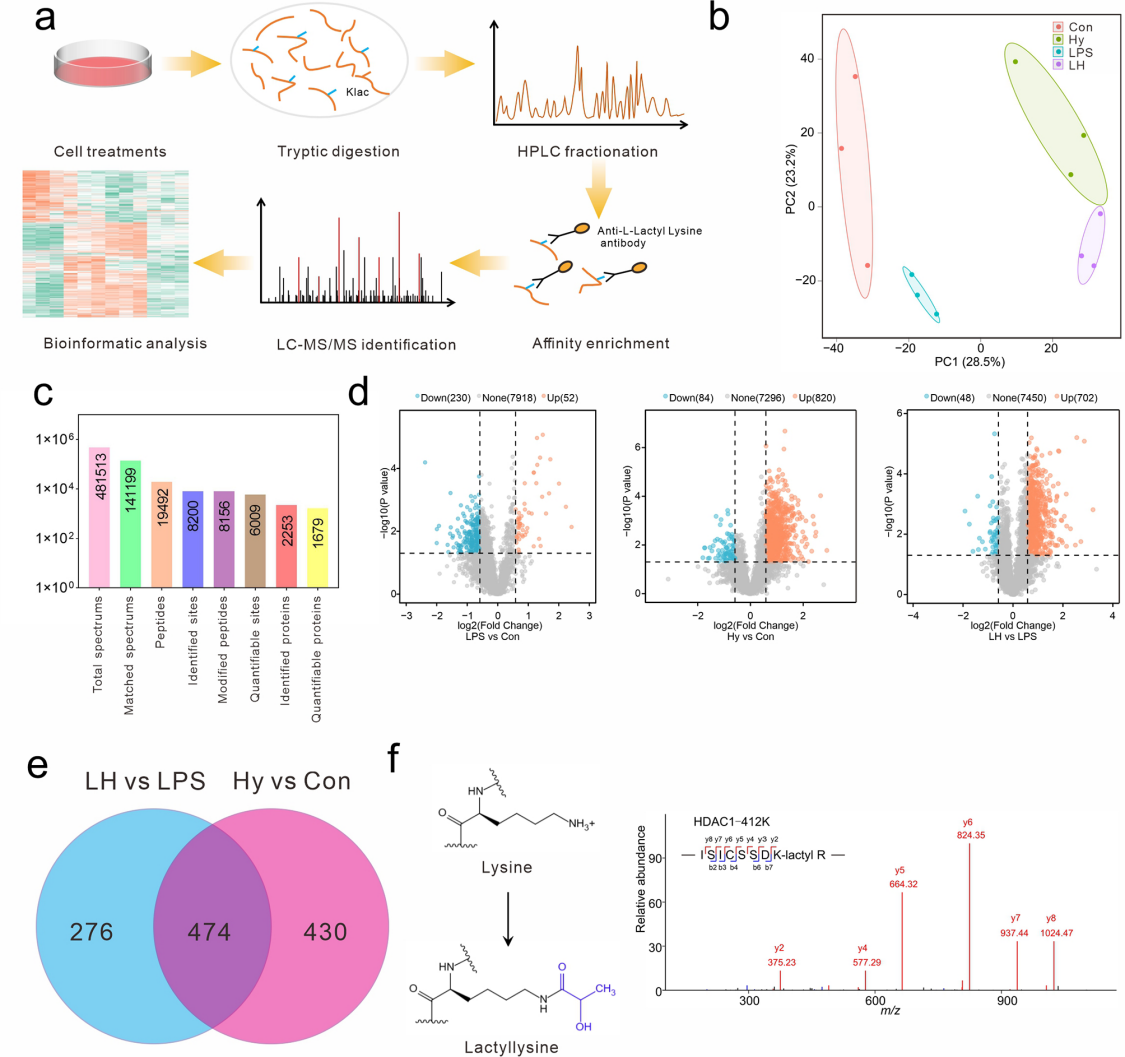
Proteomic analysis of lactylated proteins in BV-2 cells. (**a**) Schematic representation of the experimental workflow for the identification of lactylated proteins in BV-2 cells. (**b**) The plot of the principal component analysis (PCA) of the four groups. Three independent replicates in each group. (**c**) The number of identified peptides and proteins. (**d**) Volcano plots of differentiated lactyl lysine sites in the comparisons of the LPS vs. Con groups, hypoxia vs. Con groups, and LPS-hypoxia vs. LPS groups. (**e**) Venn diagram showing the overlap of lactyl lysine between the hypoxia vs. Con and LPS-hypoxia vs. LPS groups. (**f**) Representative mass spectra of lactylated peptides in the HDAC1 protein

To comprehensively elucidate protein lactylation induced by hypoxia, a proteome-wide analysis of lactylation was carried out in BV-2 cells. As shown in Fig. 3a, proteins were extracted from each group, and an anti-lactyl lysine antibody was used to enrich the lactylated peptides; these results were determined via LC‒MS/MS analysis. The similarities and differences among the samples were determined via principal component analysis (PCA). The four groups were scattered in the four areas and showed good separation of the biological replicates within the treatment samples (Fig. 3b). To visualize the overlap between lactyl sites in the triplicate repeats, UpSet plots were generated, and the repeatability of the experiments was validated (Supplementary Fig. S2). In total, 8200 lactyl sites in 2253 proteins were quantified (Fig. 3c). Compared with those in the control group, the number of lactylation sites in the LPS and hypoxia treatment groups increased by 52 and 820, respectively. There were 702 upregulated lactylation sites in the LPS/hypoxia group compared with those in the LPS group (Fig. 3d). The overlap of the differential lactylation sites between LPS/hypoxia and LPS groups and between hypoxia and Control groups showed that 474 lysine sites were lactylated (Fig. 3e). In addition, MS/MS spectra of the lactylated peptides in the HDAC1 protein were shown in Fig. 3f. Moreover, the lactylation of the HDAC1 protein was validated by immunoprecipitation and immunoblotting with an anti-pan-lactyl antibody (Supplementary Fig. S3). These results indicated that hypoxia could induce a large quantity of lactylation.

### Pattern analysis of lactylated sites

**Fig. 4:**
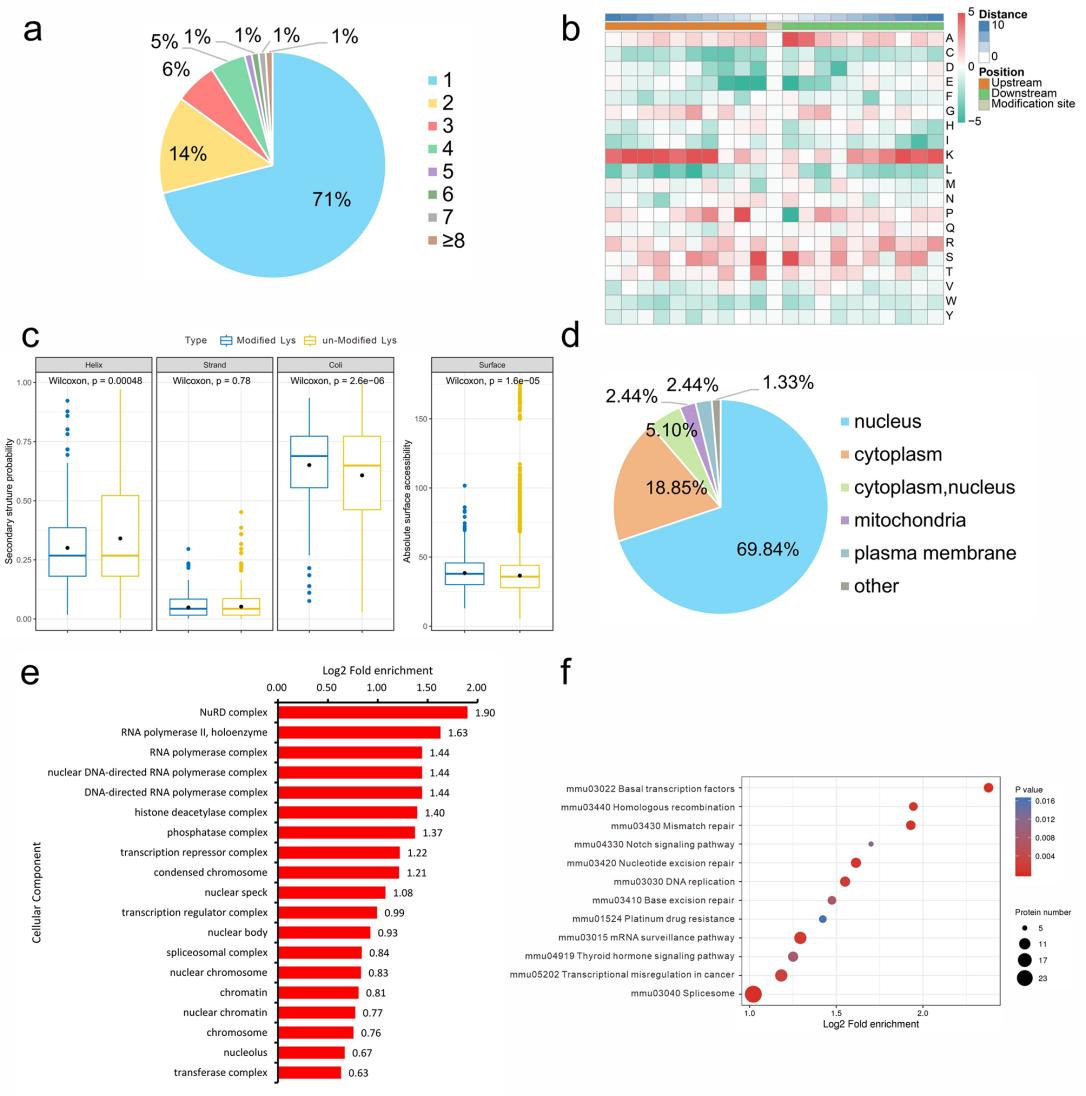
Properties of the lactylated peptides and proteins. (**a**) Distribution of lactyl lysine residues based on the number of sites per protein. (**b**) Heatmap of the amino acid compositions of lactyl lysines demonstrating the frequency of certain amino acids around the modified lysine. Red indicates a high frequency, and green indicates a low frequency. (**c**) Secondary structure distribution and surface accessibility prediction of significantly lactylated lysine sites. (**d**) Subcellular localization of upregulated differentially expressed lactylated proteins in the LPS-hypoxia group compared with the LPS group. (**e**) Distribution of upregulated differentially expressed lactylated proteins under the category of cellular component according to GO enrichment. (**f**) KEGG enrichment analysis of upregulated differentially expressed proteins

Since hypoxia can increase the neuroinflammatory response induced by LPS stimulation, the lactylation sites that differed between the LPS/hypoxia and LPS groups were subjected to comprehensive analysis. As shown in Fig. 4a, among the lactylated proteins, 71% contained a single lactyl site, 14% contained two lactyl sites, and the percentage decreased with increasing lactyl site number per protein. To understand the distribution pattern of lactylation sites, the presence of amino acids flanking the lactylated lysine sites from − 10 to + 10 was assessed. The heatmap results showed that certain amino acid residues surrounding Kla were strongly enriched. Lysine residues were enriched at the − 10 to -2, + 1, +3, and + 5 to + 10 positions (Fig. 4b). The relationship between lactylation and the secondary structures was also investigated. Among the three basic secondary structures, only the probability of a modified lysine occurring in coli was significantly greater than that of an unmodified lysine occurring. At the same time, there was also a significant difference in absolute surface accessibility when the lysine residues were lactylated (Fig. 4c). Given that hypoxia or lactate could increase the levels of lactylated proteins, the differentially upregulated lactylated proteins in the LPS/hypoxia group compared with those in the LPS group were the focus of the subsequent bioinformatics analysis. As shown in Fig. 4d, most of the lactylated proteins were distributed in the nucleus, which was consistent with the above immunofluorescence staining results (Fig. 1d). Cellular component analysis of Gene Ontology (GO) functional enrichment showed that the most enriched lactylated proteins were enriched in the NuRD complex (Fig. 4e). In support of these observations, lactylated lysine sites are mainly localized in the zinc finger domain, which is a distinguishing characteristic of transcription regulators (Supplementary Fig. S4). Consistent with these findings, KEGG pathway enrichment analysis revealed that the lactylated proteins affiliated with basal transcription factors were enriched first (Fig. 4f). These data implied that the lactylated protein may play an important role in gene transcription.

### Lactylation of protein complexes

**Fig. 5:**
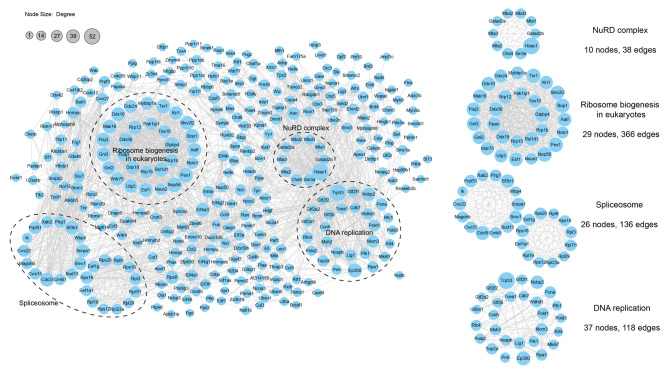
Protein-protein interaction (PPI) network analysis. Protein-protein interaction networks of upregulated differentially expressed proteins in the comparison of the LPS/hypoxia vs. LPS groups. The representative subnetworks were also shown on the right. The nodes represent the lactylated proteins, and the edges represent the interactions between lactylated proteins

To gain a better understanding of the cellular processes regulated by lactylation, a PPI network of the identified lactylated proteins was assembled using Cytoscape software. We found that several protein complexes, such as the NuRD complex, ribosome biogenesis complex, spliceosome complex, and DNA replication complex, underwent lactylation, indicating that lactylation preferentially occurred in these protein complexes (Fig. 5). Among the lactylated protein complexes, the NuRD complex is a well-known transcription regulation complex. Further modification site analysis revealed that among the core members of the NuRD complex, HDAC1, MTA1, MTA3, MTA2, MBD2, MBD3, Chd4, Gatad2α, Ncor1, Ncor2, and Sin3A all underwent lactylation, and a total of 62 lactylated sites were embedded (Supplementary Fig. S5). These results indicated that such extensive lactylation modification sites probably play an important role in the regulation of gene expression.

### The effect of lactylation on NuRD complex members

**Fig. 6:**
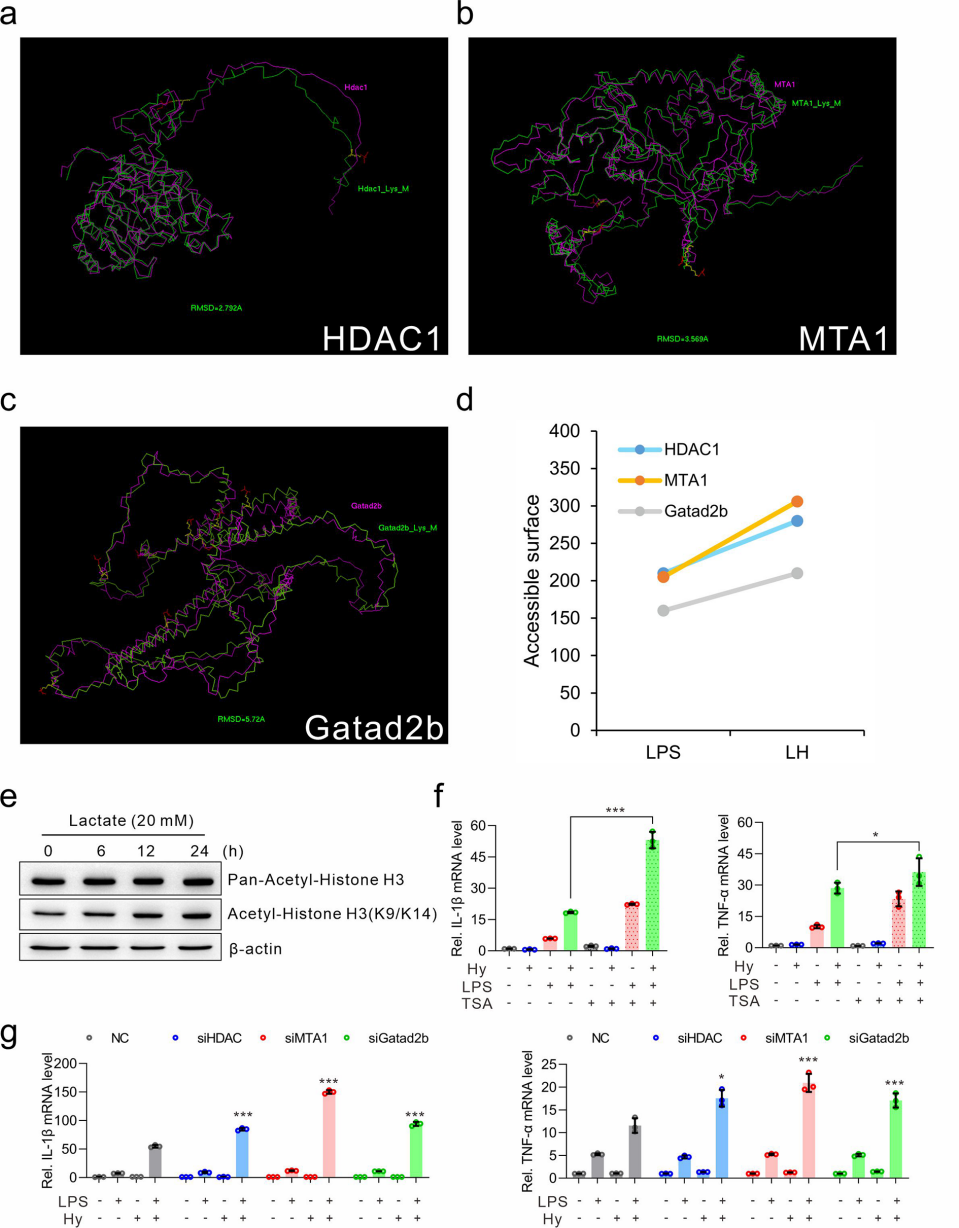
Effect of lactylation on the NuRD complex. (**a-c**) 3-D main-chain carbon atom ribbon conformations before and after lactylation of HDAC1 (Lys438, Lys473) (**a**), MTA1 (Lys462, Lys477, Lys626, Lys631) (**b**) and Gatad2b (Lys33, Lys44, Lys50, Lys52, Lys73, Lys98, Lys463, Lys499) (**c**) derived from computer-guided molecular modeling. The purple line denotes the main chain carbon atom orientation of the parent protein, and the green line denotes the lactylated proteins. The yellow balls and sticks denote the lactylated lysine residues, and the red balls and sticks denote the lactic acid molecule. (**d**) The changes in the accessibility of each protein surface were calculated via the above molecular modeling. (**e**) BV-2 cells were treated with 20 mM lactate for different time periods. The protein levels of pan-acetylated Histone H3 and acetyl-Histone H3 (K9/K14) were detected by western blotting. The expression of β-actin served as an internal control. (**f**) BV-2 cells were pretreated with 100 nM TSA for 1 h, and then treated with hypoxia (1% O_2_) alone, LPS (100 ng/ml) alone, or LH (LPS plus Hypoxia) for 24 h. The mRNA levels of IL-1β and TNF-α were detected via *q*-PCR. (**g**) BV-2 cells were transiently transfected with HDAC1-targeted siRNA (siHDAC1), MTA1-targeted siRNA (siMTA1), Gatad2b-targeted siRNA (siGatad2b), or negative control siRNA (siNC) and then treated with hypoxia (1% O_2_) alone, LPS (100 ng/ml) alone, or LH (LPS plus Hypoxia) for 24 h. The mRNA levels of IL-1β and TNF-α were detected by *q*-PCR

To test whether lactylation affects the protein structure, 3-D modeling conformations before and after lactylation modification of HDAC1 (Lys438, Lys473), MTA1 (Lys462, Lys477, Lys626, Lys631) and Gatad2b (Lys33, Lys44, Lys50, Lys52, Lys73, Lys98, Lys463, Lys499) were superimposed based on main-chain carbon atom orientation (Fig. 6a-c). Furthermore, the solvent-accessible surface area was calculated, and the 3-D theoretical structures were shown to change markedly, while the solvent accessible surface area was evidently increased (Fig. 6d). These results demonstrated that hypoxia-induced lactylation could affect the protein structure and is involved in the regulation of protein function. To test whether protein lactylation affects HDAC1 function, we treated the cells with 20 mM lactate for different time periods, and found that although pan-acetylation of histone H3 remained unchanged, specific site modifications, such as Lys9/Lys14, increased, indicating that HDAC1 activity was inhibited by exogenous lactate treatment (Fig. 6e). To test whether HDAC1 deacetylase enzyme activity contributes to cytokine expression, the HDAC1 selective inhibitor TSA was used. Inhibition of HDAC1 increased the expression of cytokines (Fig. 6f). Using RNA interference technique, we further confirmed the contribution of the NuRD complex members to the neuroinflammatory response. Knockdown of HDAC1, MTA, or Gata2b, increased the expression of TNF-α and IL-1β, indicating that these proteins play important roles in the neuroinflammatory response (Fig. 6g). Therefore, the lactylated NuRD complex is involved in increasing inflammation response under hypoxic conditions.

## Discussion

**Fig. 7:**
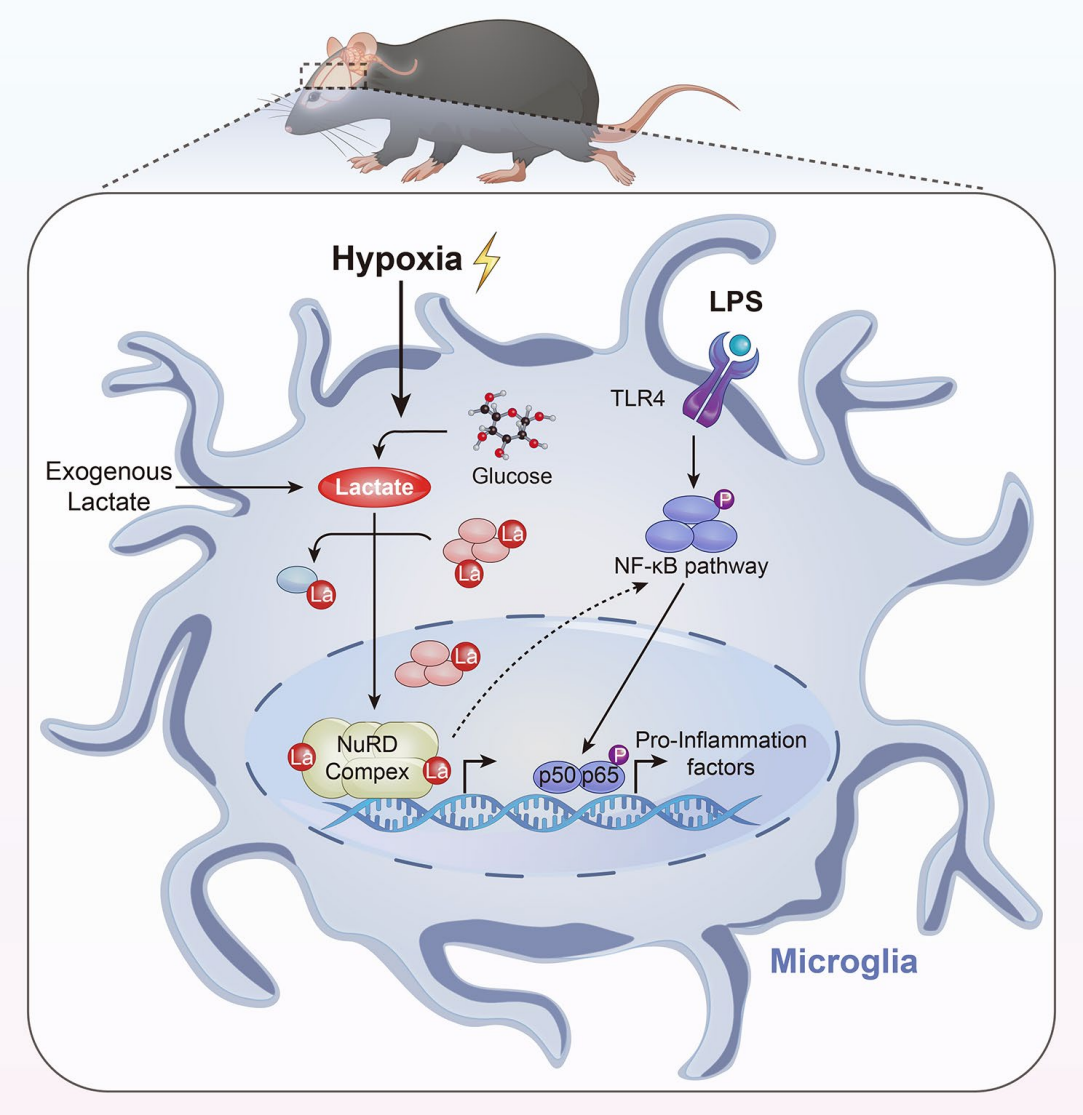
Schematic model of how hypoxia exacerbates LPS-induced neuroinflammation. In microglia, hypoxia increases the production of lactate and subsequently promotes the lactylation of proteins, which are preferentially present in protein complexes. Lactylation of the proteins in the NuRD complex changes the 3D structure of the proteins, which may result in the regulation of protein function and subsequently contribute to the inflammatory response in microglia

In the present study, we found that hypobaric hypoxia exposure increased the lactate content and lactylation modification in a mouse HACE model. Hypoxia can aggravate LPS-induced neuroinflammation in a lactate-dependent manner global lactylome analysis revealed the profiles of lactylated sites and proteins in microglia. Most of the lactylated proteins were localized in the nucleus and preferentially occurred in protein complexes, such as the NuRD complex. Lactylation modification resulted in changes in the 3D theoretical structures of the proteins (Fig. 7). These data demonstrated that lactylated proteins induced by hypoxia contribute to an increased neuroinflammatory response in experimental high-altitude cerebral edema.

Under hypoxic conditions, in addition to activating the HIF-1 signaling pathway, the key mediator of hypoxia-inducible genes, cell metabolism transits to glycolysis, thereby resulting in the production of lactate. Since it was first reported in 2019, accumulating evidence has suggested that lactate influences protein function and cellular processes by forming lysine lactylation at certain amino acid sites [9]. Lactylation of histone proteins plays an important role in macrophage homeostasis [9, 10], microglial activation [11], lung fibrosis [15], etc. Moreover, lactylation of other specific proteins has been increasingly revealed. For example, Yang et al. reported that macrophages can take up extracellular lactate to promote HMGB1 lactylation in a p300/CBP-dependent manner and subsequently impair the increase in endothelial permeability [16]. It has also been reported that Snail1 lactylation induced by lactate plays an important role in endothelial-to-mesenchymal transition following myocardial infarction [17]. In the present study, 474 proteins were identified via overlap analysis of differentially expressed proteins between the hypoxia and control groups and between the LPS/hypoxia and LPS groups; these proteins were considered to be the core lactylated proteins produced by hypoxia treatment. Protein interaction network analysis revealed that lactylation occurred within the protein complexes. In the future, it will be more interesting to identify the functions of these proteins in depth. Increasing evidence shows that lactylation of specific proteins, such as PKM2 [18], histone H3 [19, 20], and histone H4 [11], has emerged as a pivotal regulator of cell function. Moreover, the function of lactylation in different disease models, including liver injury [21], kidney fibrosis [22], gestational diabetes mellitus [23], and tumor immunity [24, 25], has been demonstrated. Therefore, targeting lactate-induced lactylation may present novel avenues for therapeutic strategies for HACE.

Neuroinflammation is closely associated with diverse neuropathologies [26]. Recent studies indicate that neuroinflammation is one of the major causes of HACE. In our previous report, increasing omics or microarray data demonstrated that inflammation and immune function are altered at high altitude and that these responses contribute to AMS [5, 6, 27]. On the other hand, the promising effect of attenuating AMS with anti-inflammatory agents, such as dexamethasone and natural compounds, further confirms that inflammation plays an important role in high-altitude cerebral edema [14, 28, 29]. Microglia are the resident macrophages in the brain and are the dominant sources of proinflammatory cytokines. Previous studies have shown that hypoxia activates microglia toward the M1 phenotype, which is associated with the release of proinflammatory cytokines [30, 31]. Here, we found that hypoxia could enhance the LPS-induced expression of cytokines and the NF-κB signaling pathway. Moreover, hypoxia exposure increased lactate production. The results of the gene knockdown and enzyme activity inhibition experiments all showed that lactate plays an important role in hypoxia-mediated aggravation of LPS-induced neuroinflammation in microglia. Previous studies have evaluated the impact of lactate on the inflammatory response in tumor-associated macrophages (TAMs). Lactate can act at multiple subcellular locations to modulate signaling and gene expression and subsequently inhibit inflammatory macrophage activation [32]. However, lactate has been reported to promote the activation of microglia, which are parallel macrophages in the brain. For example, an early report showed that lactate promoted the secretion of cytokines (TNF-α, IL-6, and IL-1β) in primary cultured microglia [33]. Liu et al. reported that lactate was beneficial for the phagocytosis, proliferation, survival, and migration of BV-2 cells [34]. Moreover, treatment of primary microglia with Deoxy-D-glucose (2-DG), an inhibitor of lactate synthesis, reduced the production of TNF-α and IL-6 through NF-κB inhibition [35]. Taken together, our findings indicate that lactate plays important roles in the regulation of microglial activation.

Chromatin remodeling is involved in the inflammatory response and contributes to differential gene expression patterns upon LPS stimulation [36, 37]. Disruption of the formation of chromatin complexes, such as by synthetic compounds, can suppress the expression of inflammatory genes [38, 39]. For instance, LPS stimulation increases PARP1 enzymatic activity and histone ADP-ribosylation in microglia and results in the accessibility of nucleosome DNA, facilitating inflammatory cytokine expression [40]. These results highlight the significance of chromatin structure for the expression of inflammatory genes. Interestingly, almost all of the members of the NuRD complex underwent lactylation. NuRD is a multi-subunit protein complex that comprises many different subunits, including the histone deacetylase HDAC1/2, ATP-dependent remodeling enzymes CHD3/4, histone chaperones RbAp46/48, CpG-binding proteins MBD2/3, GATAD2a (p66α) and/or GATAD2b (p66β) and the specific DNA-binding proteins MTA1/2/3 [41]. The NuRD complex uniquely possesses both chromatin remodeling and histone deacetylase activities and can regulate gene expression through chromatin compaction and decompaction. The significance of NuRD members in transcriptional regulation during the inflammatory response has been demonstrated by previous studies. For example, HDAC2 is recruited by Tet2 and participates in the inhibition of IL-6 gene expression [42]. The MTA2/NuRD corepressor complex negatively regulates NF-κB signaling [43]. In the present study, we found that lactylation has an impact on 3D structures, which suggests that lysine lactylation may result in the regulation of protein function and subsequently contribute to the inflammatory response in microglia.

## Conclusions

In summary, based on mouse HACE model data, we found that hypoxia treatment increased the lactate content and protein lactylation in purified microglia. Global profiling of protein lactylation in microglia revealed that lysine lactylation preferentially occurs in protein complexes and affects protein 3D structures. These data provide valuable information on the mechanism of the increased inflammatory response under hypoxic conditions. Future studies, especially studies examining the impact of lactylation on the protein function of transcription factors as well as the temporospatial pattern of protein lactylation in primary microglia, are needed to determine the roles of protein lactylation in cell function.

### Electronic supplementary material

Below is the link to the electronic supplementary material.

Supplementary Material 1

## Data Availability

No datasets were generated or analysed during the current study.

## Abbreviations

HACE: High altitude cerebral edema
2-DG: Deoxy-D-glucose
CBP: CREB-binding protein
CNS: Central nervous system
Cut&Tag: Cleavage Under Targets and Tagmentation
DMEM: Dulbecco’s minimum essential medium
GO: Gene ontology
HDAC: Histone deacetylase
HIF-1: Hypoxia-inducible factor-1
HMGB1: High-mobility group box 1
IKK: Ikappa B kinase
iNOS: Inducible nitric oxide synthase
IL-1β: Interleukin-1β
IL-6: Interleukin 6
KEGG: Kyoto encyclopedia of genes and genomes
LDH: Lactate dehydrogenase
LPS: Lipopolysaccharides
MS: Mass spectrometry
NF-κB: Nuclear factor kappa B
NuRD: Nucleosome remodeling deacetylase
PCA: Principal component analysis
PPI: Protein–protein interaction
PTGS2: Prostaglandin-Endoperoxide Synthase 2
qRT-PCR: Quantitative real-time polymerase chain reaction
SUMO: Small ubiquitin-like modifier
TAM: Tumor-associated macrophage
TNF: Tumor necrosis factor
TSS: Transcription start site
