# Supplementary material for: Global profiling of protein lactylation in microglia in experimental high-altitude cerebral edema
Source: Cell Commun Signal. 2024 Jul 25;22:374. doi: 10.1186/s12964-024-01748-x (PMC11271010; doi:10.1186/s12964-024-01748-x)
Supplement: Supplementary file 1 — Supplementary Material 1 [file 12964_2024_1748_MOESM1_ESM.docx]

Supplementary Text 1

Quality control (QC) validation of MS data and Bioinformatics analysis

1.QC Validation of MS Data

1.1. Length distribution of all identified peptides

As shown in the figure below, most of the peptides are distributed in 7-20 amino acids, which is consistent with the general rule based on trypsin enzymatic hydrolysis and HCD fragmentation. Peptides with less than 5 amino acids are too small to produce effective sequence identification. Peptides larger than 20 amino acids are not suitable for fragmentation of HCD due to their high mass and charge number. The distribution of peptide lengths identified by mass spectrometry meets quality control requirements.


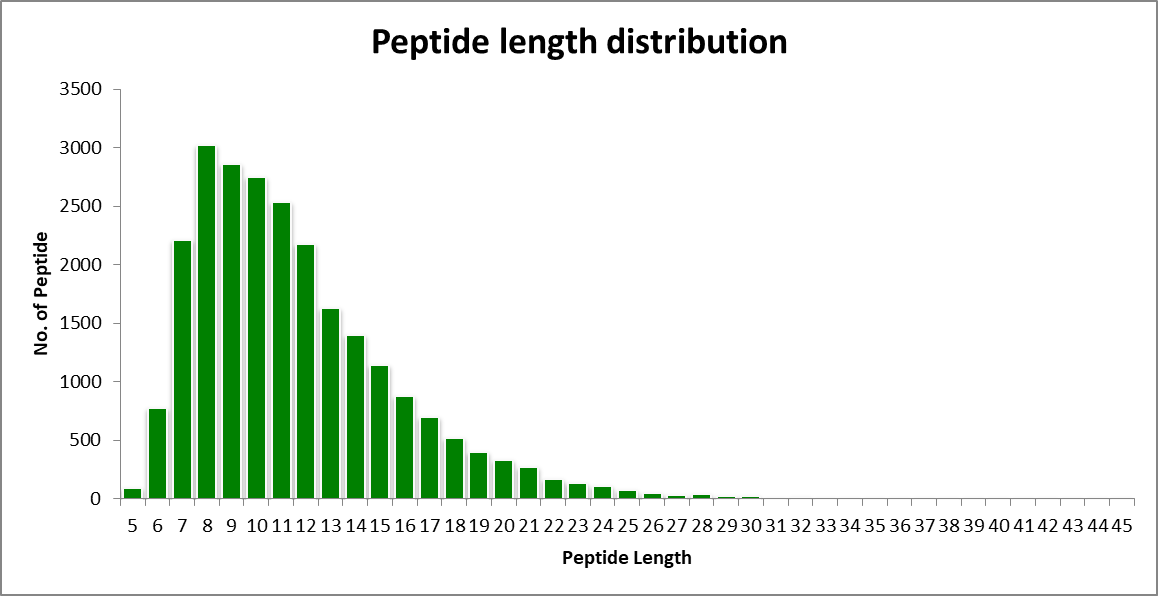


Fig.S7-1 Mass spectrometry identified the length distribution of peptides

1.2. Mass spectrometer mass tolerance distribution

As shown in the figure below, most of the spectra have a first-order mass error of less than 10 ppm, which is consistent with the high-precision characteristics of orbitrap mass spectrometry. It indicates that the mass spectrometer's mass accuracy is normal, and it will not affect the qualitative and quantitative analysis of protein due to excessive mass deviation. The score of the spectrally matched peptide (characterizing the identity of the peptide identification) was inversely related to the distribution of the mass deviation. The higher the score, the smaller the quality deviation


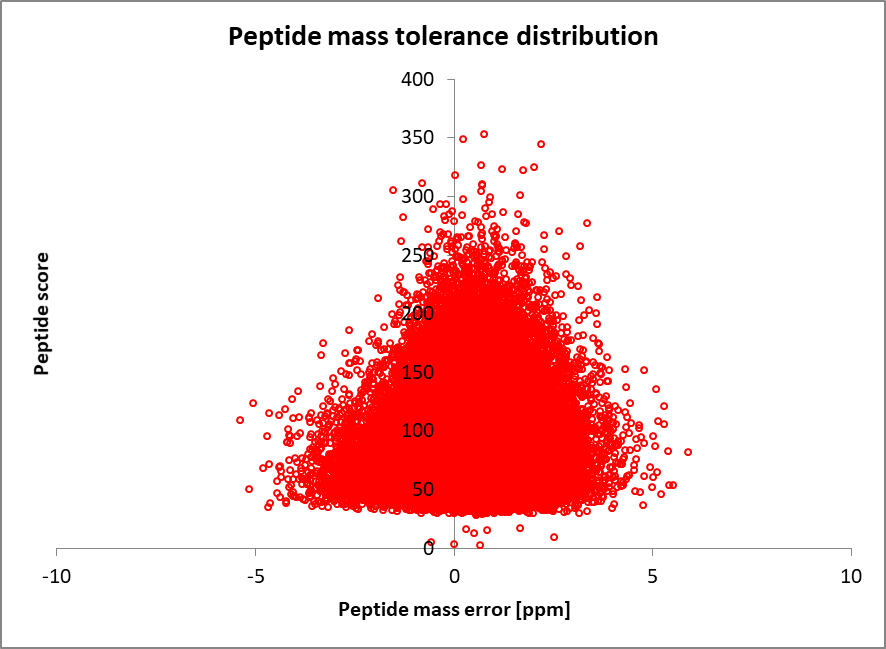


Fig.S7-2 Mass spectrometer mass tolerance distribution

2. Bioinformatics Methods

2.1 Analysis using software

Table.S7-1 software information for bioinformation analysis

| Terms | Software/Mehtod | Version/URL |
| --- | --- | --- |
| MS/MS data alignment | MaxQuant | v.1.5.2.8 <http://www.maxquant.org/> |
| Motif analysis | [MoMo](http://meme-suite.org/tools/momo) | v5.0.2 <http://meme-suite.org/tools/momo> |
| GO annotation | [InterProScan](http://www.ebi.ac.uk/interpro/) | v.5.14-53.0 <http://www.ebi.ac.uk/interpro/> |
| Domain annotation | [InterProScan](http://www.ebi.ac.uk/interpro/) | v.5.14-53.0 <http://www.ebi.ac.uk/interpro/> |
| KEGG pathway | KAAS | v.2.0 <http://www.genome.jp/kaas-bin/kaas_main> |
|  | KEGG Mapper | v2.5 <http://www.kegg.jp/kegg/mapper.html> |
| Subcellular localization | [Wolfpsort](http://wolfpsort.seq.cbrc.jp/) | v.0.2 <http://www.genscript.com/psort/wolf_psort.html> |
|  | CELLO | v.2.5 <http://cello.life.nctu.edu.tw/> |
| Enrichment analysis | Perl module | v.1.31 https://metacpan.org/pod/Text::NSP::Measures::2D::Fisher |
| Clustering heatmap | R Package pheatmap | v.2.0.3 <https://cran.r-project.org/web/packages/cluster/> |
| PPI network | Blast | v.2.2.26 <http://blast.ncbi.nlm.nih.gov/Blast.cgi> |
|  | R package networkD3 | v.0.4 https://cran.r-project.org/web/packages/networkD3/ |

### 2.2. Quantitative Analysis

The raw LC-MS datasets were first searched against database and converted into matrices containing intensity of peptides across samples. The relative quantitative value of each modified peptide was then calculated based on this intensity information by the following steps:

2.2.1. Firstly, the intensities of modified peptides (*I*) were centralized and transformed into relative quantitative values (*R*) of modified peptides in each sample. The formula is listed as follow: *i* denotes the sample and *j* denotes the modified peptide.

*Rij = Iij / Mean(Ij)*

2.2.2. If both Proteomics and Post-translational modification profiling were conducted ed on the same cohort, the relative quantitative value of the modified peptide is usually divided by the relative quantitative value of corresponding protein to remove the influence from protein expression of modifications (note: by checking whether there are protein relative expressions in 2-basic_analysis /MS_identified_information.xlsx to determine if normalization is done).

2.3. Repeatability Analysis

For experiment with biological or technical replicates, it is necessary to evaluate the quantitative reproducibility among the biological or technical replicates. Principal Component Analysis (PCA) was used to evaluate the quantitative reproducibility. Figure shows the principal component analysis results of modification sites quantitation in all samples. The closer the repeated samples from one group are aggregated, the better the quantitative repeatability is.

### 2.4. Differential Analysis

Firstly, the samples to be compared were selected in pairwise, and the fold change (*FC*) was calculated as the ratio of the mean intensity for each modification site in two sample groups. For example, to calculate the fold change between sample A and sample B, the formula is listed as following: *R* denotes the relative quantitative value of the modification site, *i* denotes the sample and *k* denotes the modification site.

*FC_A/B,k_ = Mean(R_ik_, i∈A) / Mean(R_ik_, i∈B)*

To calculate the significance of the difference between groups, student's T test was performed on the relative quantitative value of each modification site in the two sample groups, and the corresponding P value was calculated as the significance index. *P* value < 0.05 was considered as significant. To make the data conform a normal distribution, the relative quantitative value of modification site was log2 transformed. The formula is listed as following:

*P_k_ = T.test(Log2(R_ik_, i∈A), Log2(R_ik_, i∈B))*

The modification site with *P* value < 0.05, the fold change > 1.5 was regarded as significant up-regulated site, while the modification site with *P* value < 0.05, the fold change < 1/1.5 was regarded as significant down-regulated site.

2.5. Annotation Methods

GO Annotation

Gene Ontology (GO) annotation proteome was derived from the UniProt-GOA database ([www. http://www.ebi.ac.uk/GOA/](file:///C:\Users\BioPTM\Desktop\PTM_0078_iTRAQ_report\PTM_0078_iTRAQ_report\www.%20http:\www.ebi.ac.uk\GOA\)). Firstly, converting identified protein ID to UniProt ID and then mapping to GO IDs by protein ID. If some identified proteins were not annotated by UniProt-GOA database, the [InterProScan](http://www.ebi.ac.uk/interpro/) soft would be used to annotated protein’s GO functional based on protein sequence alignment method. Then proteins were classified by [Gene Ontology annotation](http://www.geneontology.org/) based on three categories: biological process, cellular component and molecular function.

Domain Annotation

Identified proteins domain functional description were annotated by [InterProScan](http://www.ebi.ac.uk/interpro/) (a sequence analysis application) based on protein sequence alignment method, and the InterPro domain database was used. InterPro (http://www.ebi.ac.uk/interpro/) is a database that integrates diverse information about protein families, domains and functional sites, and makes it freely available to the public via Web-based interfaces and services. Central to the database are diagnostic models, known as signatures, against which protein sequences can be searched to determine their potential function. InterPro has utility in the large-scale analysis of whole genomes and meta-genomes, as well as in characterizing individual protein sequences.

KEGG Pathway Annotation

[Kyoto Encyclopedia of Genes and Genomes (KEGG)](http://www.genome.jp/kegg/) database was used to annotate protein pathway. Firstly, using KEGG online service tools KAAS to annotated protein’s KEGG database description. Then mapping the annotation result on the KEGG pathway database using KEGG online service tools KEGG mapper.

Subcellular Localization

There, we used [wolfpsort](http://wolfpsort.seq.cbrc.jp/) a subcellular localization predication soft to predict subcellular localization. [Wolfpsort](http://wolfpsort.seq.cbrc.jp/) is an updated version of PSORT/PSORT II for the prediction of eukaryotic sequences. Special for protokaryon species, Subcellular localization prediction soft CELLO was used.

2.6 Functional Enrichment

Enrichment of Gene Ontology analysis

Proteins were classified by GO annotation into three categories: biological process, cellular compartment and molecular function. For each category, a two-tailed Fisher’s exact test was employed to test the enrichment of the differentially modified proteins against all identified proteins. The GO with a corrected p-value < 0.05 is considered significant.

Enrichment of pathway analysis

Encyclopedia of Genes and Genomes (KEGG) database was used to identify enriched pathways by a two-tailed Fisher’s exact test to test the enrichment of the differentially modified proteins against all identified proteins. The pathway with a corrected p-value < 0.05 was considered significant. These pathways were classified into hierarchical categories according to the KEGG website.

Enrichment of protein domain analysis

For each category proteins, InterPro (a resource that provides functional analysis of protein sequences by classifying them into families and predicting the presence of domains and important sites) database was researched and a two-tailed Fisher’s exact test was employed to test the enrichment of the differentially modified proteins against all identified proteins. Protein domains with a p-value < 0.05 were considered significant.

2.7. Enrichment-based Clustering

For further hierarchical clustering based on different protein functional classification (such as: GO, Domain, Pathway, Complex). We first collated all the categories obtained after enrichment along with their P values, and then filtered for those categories which were at least enriched in one of the clusters with P value <0.05. This filtered p value matrix was transformed by the function x = −log10 (P value). Finally, these x values were z-transformed for each functional category. These z scores were then clustered by one-way hierarchical clustering (Euclidean distance, average linkage clustering) in Genesis. Cluster membership were visualized by a heat map using the “heatmap.2” function from the “gplots” R-package.

2.8. Protein-protein Interaction Network

All differentially modified proteins database accession or sequence were searched against the STRING database version 10.5 for protein-protein interactions. Only interactions between the proteins belonging to the searched data set were selected, thereby excluding external candidates. STRING defines a metric called “confidence score” to define interaction confidence; we fetched all interactions that had a confidence score >0.7 (high confidence). Interaction network form STRING was visualized in R package “networkD3”.

2.9. Motif Analysis

Soft [MoMo](http://meme-suite.org/tools/momo) (motif-x algorithm) was used to analysis the model of sequences constituted with amino acids in specific positions of modify-21-mers (10 amino acids upstream and downstream of the site， but phosphorylation with modify-13-mers that 6 amino acids upstream and downstream of the site) in all protein sequences. And all the database protein sequences were used as background database parameter. Minimum number of occurrences was set to 20. Emulate original motif-x was ticked, and other parameters with default.

**Fig. S1 Determination of the efficiency of LDHA knockdown**

LDHA-targeted siRNA (LDHA-siRNA) or negative control siRNA (NC-siRNA) were synthesized by RiboBio (Guangzhou, China), and their sequences are as following:

| Oligo name | Sequence |
| --- | --- |
| LDHA-siRNA | GAACAAGAUUACAGUUGUUdTdT |
| NC-siRNA | UUCUCCGAACGUGUCACGUdTdT |

The knockdown efficiency was determined by western blot, which probbed with LDHA primary antibody (Cat:3582, Cell Signaling Technology).


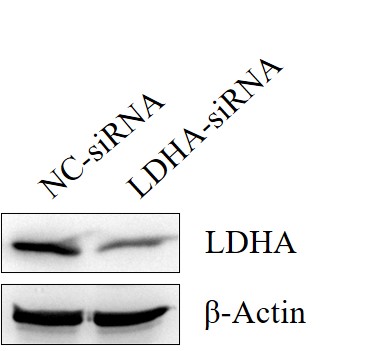


Fig. S2 Number of lactyl lysie sites quantified in three biological replicates of each group.

**
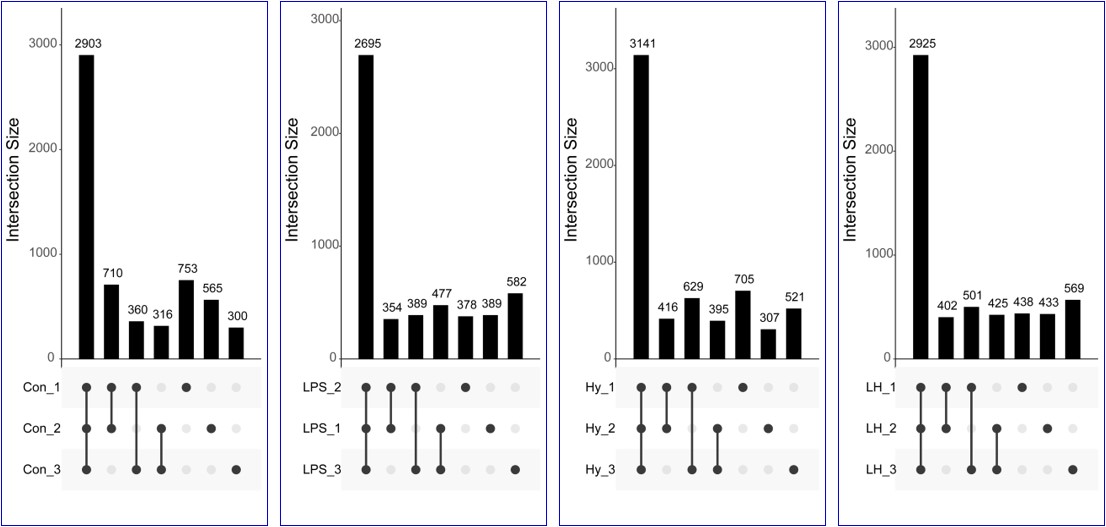
**

Fig. S3 Validation of protein lactylation

BV-2 cells (A) or 293 cells which were transiently transfected flag-HDAC1 plasmid (B) were treated with hypoxia (1% O2) for 24 h. Cell lysates were immunoprecipitated with the indicated primary antibody (anti-HDAC1, Cat:34589, Cell Signaling Technology; anti-Flag, Cat:14793, Cell Signaling Technology) and analyzed by immunoblotting with anti-pan lactyl lysine antibody.


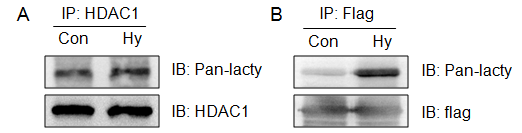


Fig. S4 Domain analysis of differential lactylated proteins between LPS/hypoxia and LPS groups


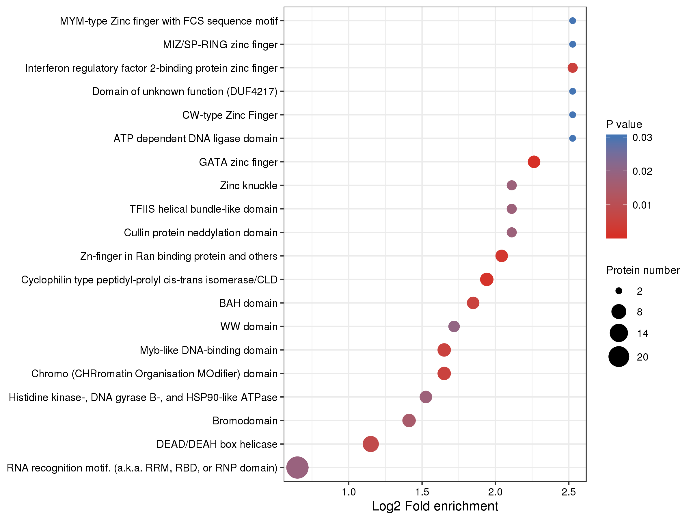


Fig. S
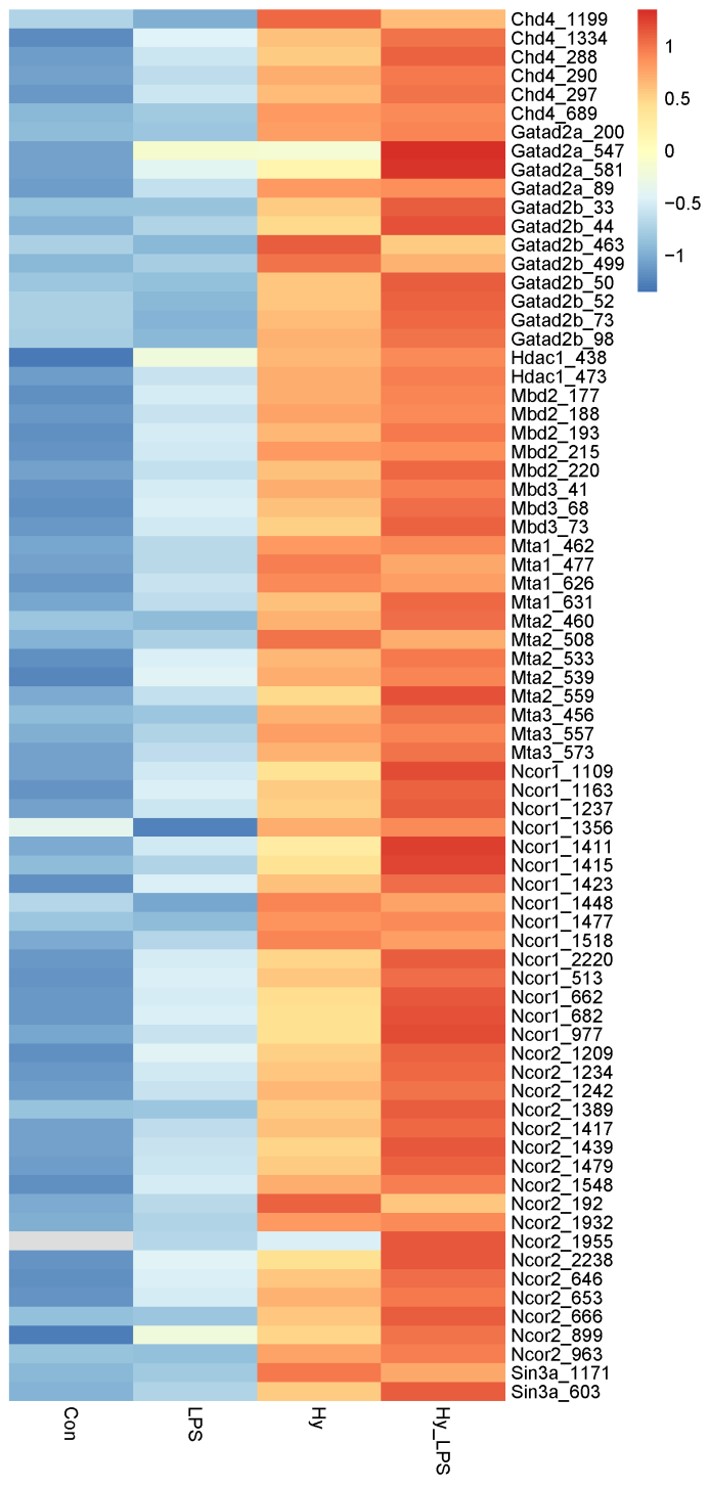
5 The lactylation sites in the protein members of NuRD complex

**Table. S1 The sequence of primers in *q*-PCR assay**

| **Gene names** | **Sequences** |
| --- | --- |
| IL-6 | F: agttgccttcttgggactga |
|  | R: tccacgatttcccagagaac |
| IL-1β | F: gggcctcaaaggaaagaatc |
|  | R: taccagttggggaactctgc |
| TNF-α | F: tatggctcagggtccaactc |
|  | R: ctccctttgcagaactcagg |
| iNOS | F: caccttggagttcacccagt |
|  | R: accactcgtacttgggatgc |
| β-Actin | F: gtgggaatgggtcagaagga |
|  | R: cttctccatgtcgtcccagt |

**Table. S2 The sequence of siRNA targeting HDAC1, MTA1 and Gatad2b**

| **Gene names** | **Sequences** |
| --- | --- |
| HDAC1 | GCTTCTGTTACGTCAATGAdTdT |
| MTA1 | GACCAAACCGCAATAACATdTdT |
| Gatad2b | GGACAACAAGGCTTATCTAdTdT |
